# The identification and expression analysis of walnut Acyl-ACP thioesterases

**DOI:** 10.3389/fgene.2024.1409159

**Published:** 2024-07-29

**Authors:** Hui Wang, Jianqing Shi, Wanhui Guo, Xiaohui Sun, Shuhui Niu, Li Chen, Shenghong Liu, Lei Ma

**Affiliations:** ^1^ Key Laboratory of Agro-Products Quality and Safety of Xinjiang, Institute of Agricultural Quality Standards and Testing Technology, Xinjiang Academy of Agricultural Sciences, Ürümqi, China; ^2^ Jiepin Planting Farmers’ Professional Cooperative, Maigaiti, China; ^3^ College of Food Science and Pharmacy, Xinjiang Agricultural University, Ürümqi, China

**Keywords:** walnut, fatty acid, Acyl-ACP thioesterase, thioesterase, expression pattern

## Abstract

Walnuts (*Juglans regia* L.), renowned for their nutritional potency, are a rich source of unsaturated fatty acids. Their regular intake plays a pivotal role in health maintenance and recuperation from a myriad of ailments. Fatty acyl-acyl carrier protein thioesterases, which orchestrate the hydrolysis of acyl-ACP thioester bonds, thereby yielding fatty acids of varying chain lengths, are instrumental in augmenting plant fatty acid content and modulating the balance between saturated and unsaturated fatty acids. Despite some investigative efforts into the synthesis and metabolic pathways of fatty acids in walnuts, our comprehension of Fat in walnuts remains rudimentary. This research undertook a comprehensive characterization of the JrFat family, predicated on the complete genome sequence of walnuts, leading to the identification of 8 JrFat genes and an exploration of their protein physicochemical properties. Utilizing *Arabidopsis* and soybean Fat genes as outgroups, JrFat genes can be categorized into 5 distinct subgroups, three of which encompass a pair of homologous gene pairs. These genes have demonstrated remarkable conservation throughout the evolutionary process, with highly analogous conserved base sequences. The promoter region of JrFats genes predominantly harbors light response and plant hormone response regulatory elements, with no discernible disparity in promoter elements among different JrFats. Predictive analyses indicate that JrFats proteins engage extensively with walnut fatty acid synthesis and metabolism-associated proteins. qRT-PCR analysis reveals an initial surge in the expression of JrFats during the development of walnut kernels, which either stabilizes or diminishes following the hard core period. Homologous gene pairs exhibit analogous expression patterns, and the expression trajectory of JrFats aligns with the dynamic accumulation of fatty acids in kernels. The expression of *JrFatA2* exhibits a strong correlation with the content of Alpha-linolenic acid, while the expression of *JrFatB2* is inversely correlated with the content of two saturated fatty acids. Collectively, these findings enrich our understanding of fatty acid synthesis and metabolism in walnuts and furnish gene resources for enhancing the content and ratio of fatty acids in walnuts.

## 1 Introduction

Walnuts (*Juglans regia* L.), arboreal nuts of antiquity, are globally propagated and esteemed for their distinctive flavor and copious nutritional composition. A trifling ounce of walnut kernels proffers a plethora of essential minerals, proteins, fibers, vitamins, and a significant proportion of fats, primarily healthful polyunsaturated fats (Vinson and Cai, 2012). The lipid constitution of walnuts is notably exceptional and beneficial. They are among the rare plant-derived sources of omega-3 fatty acids, particularly ALA, which constitutes approximately 13% of their total fat content, contributing significantly to cardiovascular health (Kris-Etherton et al., 2008). Furthermore, walnuts are rich in monounsaturated fatty acids (MUFAs), predominantly oleic acid, which is associated with improved lipid profiles and reduced risk of cardiovascular diseases. Polyunsaturated fatty acids (PUFAs), such as linoleic acid, are also abundant, further enhancing the health-promoting attributes of walnuts (Mukuddem-Petersen et al., 2005; Tsamouris et al., 2002; Beyhan and Michailides, 2005).

Plant seeds undergo intricate biochemical processes to accomplish *de novo* synthesis of fatty acids (FAs), primarily within the stroma of plastids and the endoplasmic reticulum, involving a multitude of enzymes and metabolic pathways (He et al., 2020). Fatty acyl–acyl carrier protein thioesterases (Fats, EC 3.1.2.14) are pivotal enzymes in plant fatty acid synthesis and metabolism. They belong to the Thioesterases (TEs) enzyme family, characterized by two helix/multi-stranded sheet motifs, known as hotdog domains. The highly conserved N-terminal domain of these enzymes is primarily involved in substrate recognition specificity, while the C-terminal domain participates in catalytic reactions (Mayer and Shanklin, 2005). Fat enzymes catalyze the hydrolysis of acyl-ACP thioester bonds, thereby terminating fatty acid synthesis and releasing free fatty acids and acyl–acyl carrier protein (ACP) into the cytosol. Different Fats recognize different types of fatty acids through substrate-specific recognition, thereby modulating fatty acid chain length, saturation, and composition. In plants, Fats are broadly classified into FatAs and FatBs (Voelker et al., 1997). FatA enzymes preferentially utilize unsaturated fatty acids as substrates, exhibiting higher activity towards 18:1-ACP. FatBs exhibit extensive activity towards unsaturated fatty acids (mainly palmitic acid 16:0 and stearic acid 18:0), with subgroup 1 FatBs showing a preference for long-chain fatty acids, while subgroup 2 FatBs prefer medium-length chain fatty acids (Zhou et al., 2021; Liu et al., 2022a; Peng et al., 2020). While the specificity of different subfamilies of Fat enzymes exhibits certain preferences, this is not absolute. The acyl-binding cavity of the Fat enzyme to some extent determines the type of fatty acid. Smaller acyl-binding cavity can accommodate shorter acyl chains, whereas larger ones tend to produce long-chain fatty acids (Feng et al., 2017). Moreover, the interaction between Fat and ACP can also influence the chain length and yield of fatty acid synthesis. The substitution of nonpolar amino acids on the surface of Fat has led to a more than threefold increase in the content of medium-chain fatty acids in *E. coli* (Sarria et al., 2018).

Genetic investigations have corroborated that the expression of Fat genes can markedly modulate the content and composition of fatty acids (FAs). For instance, *Arabidopsis thaliana* harbors two FatA genes and a solitary FatB gene. In the fata1 and fata2 double mutants, the expression levels of *AtFatA1* and *AtFatA2* diminish by more than half, culminating in a reduction in unsaturated fatty acids 18:0, 18:1, and 18:2 in desiccated seeds (Moreno-Pérez et al., 2012). Conversely, in specimens overexpressing *AtFatB1* driven by a heterologous promoter, there is a pronounced augmentation in the content of 16:0, 18:0, and 14:0 fatty acids in seeds, while the content of unsaturated fatty acids (including 18:1, 18:2, 18:3, 20:1, and 22:1) significantly decreases (Dörmann et al., 2000). Moreover, alterations in AtFat gene expression levels also precipitate growth inhibition, modifications in seed number and size, as well as abnormal seed morphology and vigor in *Arabidopsis* (Bonaventure et al., 2003). Mutations in *GmFatA1A* in soybean can escalate oleic acid content by 34.5%, while mutations in *GmFatB* result in a 5.6% reduction in palmitic acid content (Zhou et al., 2023). Overexpression of four GmFatB genes in *Arabidopsis* led to a significant increase in the content of palmitic acid and stearic acid, resulting in an enhancement of seed oil content by 7.5%–12.5% (Zhao et al., 2024). The expression patterns of GhA-FatB3 and *GhD-FatB4* in cotton are positively correlated with the dynamic accumulation of C16:0 in seeds, with significant augmentations in palmitic acid content in overexpressing leaves (Liu et al., 2022a). Overexpression of the *EgFatB1* gene in Elaeis guineensis significantly amplifies the content of C16:0 in seeds from approximately 8% to around 33% (Xia et al., 2019). In yeast cells overexpressing the *BnFatB* gene in rapeseed, the content of C16:0 and C18:0 surges by 45.7% and 21.7%, respectively, while C16:1 and C18:1 decrease by 15.3% and 30.6%, respectively (Tan et al., 2015). Furthermore, the overexpression of Fat to facilitate the release of free fatty acids from the FAS system is one of the significant methods in synthetic biology to augment fatty acid titers (Jing et al., 2018). Both traditional and machine learning approaches also have been employed to investigate the substrate activity preferences and catalytic mechanisms of Fat (Jing et al., 2024).

Mature walnut kernels, affluent in fatty acids, with polyunsaturated fatty acids constituting over 70% of their composition, are an unparalleled source of unsaturated fatty acids. Numerous studies have probed into and deliberated key genes implicated in fatty acid synthesis and metabolism in different walnut kernels, such as FAD (fatty acid desaturase), OLE (oleosin), and SAD (stearoyl-ACP desaturase) (Huang et al., 2021; Zhou et al., 2023; Shi et al., 2022). However, the role of Fats as pivotal enzymes in walnut saturated and unsaturated fatty acid synthesis and compositional alterations remains largely uncharted. The advancement of walnut genomics has facilitated the comprehensive characterization of all walnut Fat genes at the whole-genome level. In this study, we identified two FatA genes and six FatB genes in walnuts, exploring their gene structures, conserved motifs, phylogeny, homogeneity, expression regulation, and protein interactions. Additionally, we quantified the expression of Fat genes in walnut kernels and investigated their correlation with the accumulation of different fatty acids. These findings deepen our understanding of walnut fatty acid metabolism and regulatory networks, while also providing genetic resources for adjusting and optimizing the fatty acid content and proportion in walnut kernels.

## 2 Materials and methods

### 2.1 Plant material and fatty acid content determination

The walnut material used in this study is “XinXin2” from the Ministry of Agriculture and Rural Affairs’ Fruit Tree Science Observation Station in Xinjiang (Yecheng County, Kashgar). From July to September 2023, fruits of ten-year-old walnuts were collected at different stages: rapid growth period (S1, S2), hard core period (S3), rapid oil conversion period (S4), and fruit maturity period (S5). During collection, 30 fruits were selected each time from the middle of the canopy periphery of five test plants, choosing fruits of equal size, without injury or pest damage. After separating and removing the clean pulp, the kernels were quickly frozen in liquid nitrogen and stored in cryopreservation tubes for subsequent gene expression quantification. At the same time, gas chromatography was used to determine the fatty acid content in fruits at different stages. The sample processing and measurement methods refer to previous reports (Liu et al., 2022a).

### 2.2 Identification and characterization of fat genes

*Arabidopsis* and soybean Fats pprotein were used as query sequences to blastp to the whole genome sequence of walnut to screen for candidate members. The E-value threshold for alignment was set to 1e-5. Simultaneously, the HMM model of the Acyl-ACP thioesterase N-terminal domain (PF01643) was employed to conduct a genome-wide search in walnut, with the threshold set to an E-value < 1e-5. The intersection of the two search results was considered as the candidate members. For the screened members, the sequences were manually submitted to the CDD (http://blast.ncbi.nlm.nih.gov) and SMART (http://smart.embl-heidelberg.de/) databases to remove genes with incomplete structure and without conservative domains. The remaining genes were identified as members of the walnut Fat gene family and named. The sequences were submitted to the ExPASy (http://web.expasy.org/compute_pi/) database to calculate the physicochemical properties of the protein, and the WoLF PSORT website (https://wolfpsort.hgc.jp/) was used to predict the subcellular localization of JrFats.

### 2.3 Gene structure and motif analysis

The annotation information of JrFat was manually extracted from the walnut genome annotation file. The MEME website (http://meme-suite.org/) was used to search for conservative base sequences of JrFat, with the minimum motif length set to 6, the maximum motif length set to 50, and the number of motifs set to 10. A phylogenetic tree of JrFat was constructed using MEGA7, using the neighbor-joining method, and bootstrap was set to 1,000. All results were visualized through Tbtools.

### 2.4 Phylogenetic tree and homology relationship analysis

A phylogenetic tree of Fats genes from *Arabidopsis*, soybean, and walnut was constructed using the IQtree software, using the maximum likelihood method, and bootstrap was set to 1,000. MscanX was used to separately screen for the paralogous homology relationship of walnut JrFats genes and their orthologous homology relationship with *Arabidopsis* and soybean, to judge the replication type of JrFat homologous gene pairs. The KaKs_caculate software (v3) was used to calculate the nonsynonymous substitutions per nonsynonymous site (Ka) and the number of synonymous substitutions per synonymous site (Ks) values between homologous gene pairs (Zhang, 2020), and the divergence time of gene pairs was calculated using the chromosome rearrangement rate (1.30E-08).

### 2.5 Promoter element and interacting protein analysis

The upstream 2,000 bp sequence of each *JrFats* gene was extracted as its promoter sequence and submitted to the PlantCARE database (https://bioinformatics.psb.ugent.be/webtools/plantcare/html/) to predict the *cis*-acting elements (CREs) in the promoter region. At the same time, the protein sequence of JrFats was submitted to the STRING database (https://string-db.org/) to search for and predict protein interaction relationships, and the protein interaction network was constructed using Cytoscape software.

### 2.6 Real-time fluorescence quantification and correlation analysis

The kernels of walnuts at different developmental stages were thoroughly crushed and ground after being frozen in liquid nitrogen, and then the EASYspin Plant RNA Rapid Extraction Kit (Beijing Tiangen Biotech Co., Ltd., Beijing, China) was used to extract RNA from the kernels according to the standard procedure provided. Agarose gel electrophoresis was used to determine the integrity and concentration of RNA, and then the qualified RNA was reverse transcribed into cDNA. qRT-PCR primers were designed for JrFats genes, and the primer list can be seen in the Supplementary Table S1. The primers were synthesized by Sangon Biotech Co., Ltd. (Shanghai, China). Using walnut 18S as an internal reference gene, the reaction was carried out using the Bio-rad CFX96 Real Time System (Hercules, California, United States) according to the standard procedure of the TAKARA SYBR Premix Ex Taq kit. Each experiment included three technical replicates and three biological replicates, and the expression level was calculated using the 2^−ΔΔCt^ technique. Visualizations and significance calculations were performed using Prism software (*p* < 0.01). The correlation between expression levels and fatty acid content was calculated using the Pearson correlation coefficient in R.

## 3 Results

### 3.1 Identification of walnut fat gene

To identify Fat genes in walnuts, the amino acid sequences of Fat genes from *Arabidopsis* and soybean were utilized as query sequences for a blastp analysis against the walnut genome. Genes with high recognition rates were further scrutinized for domain determination, culminating in the identification of eight genes as members of the walnut Fat gene family (Table 1). The Fat gene was initially classified into the FatA/B subfamilies based on blast results, subsequently, they were named as JrFatA1-JrFatA2 and JrFatB1-JrFatB6 according to their respective physical loci in the genome. The 8 JrFat genes are dispersed across seven chromosomes. Apart from *JrFatB3*, which has a shorter amino acid length (223 aa) and lower molecular weight (25,683.22), the remaining JrFat amino acid lengths range from 379 to 459, and their molecular weights range from 42,686.53 to 51,806.35. The pI values of JrFat span widely from 6.5 to 9.41, indicating that all JrFat proteins are hydrophobic proteins. Predictive results suggest that *JrFatA* proteins may localize to the chloroplast, while *JrFatB* proteins may localize to the nucleus, cytoplasm, and chloroplast.

**TABLE 1:** Characterization and physicochemical properties of all JrFats.

Gene name	Chr	Start	End	Protein length	MW	pI	Gravy	Subcell
JrFatA1	7	5,588,223	5,593,956	385	43,102.1	7.1	−0.365	chlo
JrFatA2	12	3,239,526	3,247,986	379	42,686.53	6.5	−0.39	chlo
JrFatB1	1	39,698,635	39,704,427	418	46,182.59	6.6	−0.296	chlo
JrFatB4	2	722,892	725,989	389	44,045.16	9.3	−0.442	cyto
JrFatB2	10	32,584,696	32,589,057	432	47,609.39	6.6	−0.234	chlo
JrFatB3	12	27,572,777	27,574,700	223	25,683.22	6.1	−0.371	nucl
JrFatB5	13	37,397,205	37,408,634	459	51,806.35	9.4	−0.429	chlo
JrFatB6	16	2,524,501	2,526,947	404	45,600.64	8.6	−0.503	nucl

### 3.2 Phylogenetics, motif and structure analysis of JrFat gene

**FIGURE 1:**
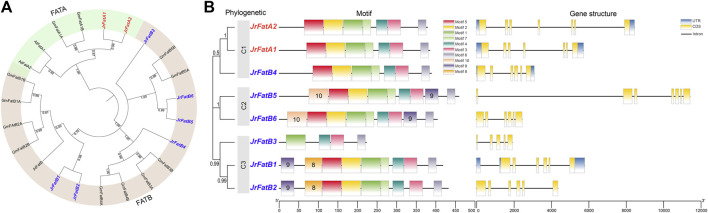
Phylogenetic, ggene structure and motif analysis of *JrFats*. **(A)** Phylogenetic tree of Fats between species, with *JrFats* indicated by red labels and numbers at branches representing bootstrap values. *FatA* and *FatB* are displayed in different colors. **(B)** The numbers in the phylogenetic tree section represent bootstrap values.

To further elucidate the evolutionary status and relationships of JrFat, we incorporated the Fat genes from *Arabidopsis* thaliana and soybean as outgroups to construct a phylogenetic tree (Figure 1A). Almost all branches obtained high confidence that the tree is stable. All 23 fat genes from the three species were classified into two subfamily, FATA and FATB. The number of FatA is identical across the three species, all of which reside within the same subgroup. In contrast, the FatB subfamily exhibits divergence among the three species. *JrFatB1* and *JrFatB2* clustered together with *AtFatB* from *Arabidopsis* thaliana and *GmFatB1/B2* from soybean, forming a clade that likely represents the most conserved members within the FatB family. Only one *JrFatB4* grouped with four *GmFatB* in the same clade, whereas *AtFatB* is absent, suggesting functional divergence or loss within this subgroup in walnuts, leading to a contraction of family members within this subgroup. *JrFatB5/6* and *GmFatB5A/5B* are positioned on the same branch, with *Arabidopsis* similarly absent within this grouping. *JrFatB3* displayed distinctiveness, standing apart from all other *FatB* genes, suggesting its unique phylogenetic position and sequence characteristics may be associated with specific functions.

A fundamental gene feature analysis was conducted on the JrFat gene family (Figure 1B). In the phylogenetic tree of Fat within the walnut, JrFat was divided into three clades (C1-C3). *JrFatA1* and *JrFatA2* both belong to C1, although *JrFatB4* is also assigned to this clade despite a relatively low bootstrap value. *JrFatB5* and *JrFatB6* are situated in C2, while the remaining 3 *JrFatB* members are in C3. Although members of the JrFat gene family vary in length, their gene structures are relatively simple. All JrFat genes have 5 to 7 exons, with only *JrFatA1*/*A2/B4/B1* possessing complete UTRs. *JrFatB5* has the longest gene due to a single long intron, while *JrFatB3* has the shortest gene. There appear to be no structural differences between *JrFatA* and *JrFatB* genes. Regarding conserved motifs, almost all JrFat genes exhibit similar conserved sequences, with motifs 1–7 distributed in the middle and C-terminal regions of the genes. However, *JrFatB3* is an exception once again, reflecting its shorter amino acid length and possession of only four motifs, consistent with its gene and protein structures. Minor differences in motif distribution exist between JrFat genes in different clades. Specifically, motifs 8 and 10 are uniquely present in C3 (except *JrFatB3*) and C2, respectively, while C1 lacks unique motifs. Additionally, the distribution of motif nine differs between clades, potentially reflecting differences in the localization and substrate recognition of different JrFat proteins.

### 3.3 Paralogs and orthologs analysis of JrFat gene

**FIGURE 2:**
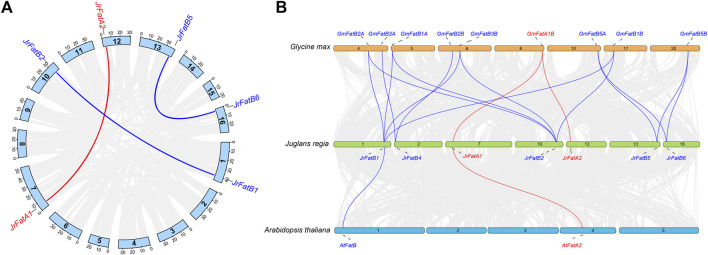
Homology analyses of *JrFats*. **(A)** Paralogous homology of *JrFats*. *JrFatA* and *JrFatB* are shown in different colours, and the connecting lines represent genes that are paralogous to each other. **(B)** The Orthology of Fats genes in walnuts arabidopsis and soybeans is depicted. *FatA* and *FatB* are displayed in different colors, and lines represent Orthologous relationship.

The homologous relationships of JrFat are investigated. Within the JrFat gene family, there are three pairs of paralogs: *JrFatA1-JrFatA2*, *JrFatB1*-*JrFatB2*, and *JrFatB5*-*JrFatB6*, all of which cluster together in the phylogenetic tree (Figure 2A). *JrFatB3* and *JrFatB4* lack paralogs (Figure 2A). Additionally, utilizing whole-genome alignment, orthologs between walnut, model plants *Arabidopsis thaliana*, and soybean, the latter being a high-oil content species with a well-characterized Fat, have been screened. Except for *JrFatB3*, the remaining 7 JrFat genes exhibit direct orthologous relationships with *GmFat* genes (Figure 2B). *JrFatB1*/*B2* each have four orthologs, namely *GmFatB1*/*B2* genes, maintaining both evolutionary conservation and orthologous relationships. *JrFatB4* has two orthologs, *GmFatB3A* and *GmFatB3B*, indicating potential gene loss or functional divergence of its paralogs, similar to the situation observed for *JrFatB3*, which lacks both paralogs and orthologs. *JrFatA1/A2* are only orthologous to *GmFatA1B*, with no direct orthologous relationship with *GmFatA1A*, despite their high sequence similarity and placement within the same branch of the phylogenetic tree, suggesting that the *JrFatB* subgroup may be more evolutionarily conserved than the *JrFatA* subgroup.

### 3.4 Synonymous and nonsynonymous substitution rates analysis of JrFat gene

We calculated the ratio of Ka to Ks for the three pairs of paralogs within the JrFat gene family (Table 2). The Ka/Ks ratios for *JrFatA1-JrFatA2*, *JrFatB1-JrFatB2*, and *JrFatB5-JrFatB6* were 0.18, 0.15, and 0.21, respectively, all significantly below 1. This indicates strong negative selection acting on these gene pairs, resulting in protein sequences that tend to be stable with slow evolutionary rates. All gene pairs are located on different chromosomes, suggesting segmental duplication. Furthermore, we estimated the divergence times between different gene pairs. *JrFatA1-JrFatA2* diverged approximately 15.71 million years ago (Mya), *JrFatB1*-*JrFatB2* diverged approximately 14.04 Mya, and *JrFatB5-JrFatB6* diverged approximately 13.18 Mya. The divergence times of JrFat genes are relatively close to each other and significantly more recent than the divergence time between *J. regia* and *M. rubra* (31 Mya) and the last whole genome duplication event (24.48 Mya). However, they are considerably earlier than the divergence time between *J. regia* and its related species (at the latest 3.45 Mya) (Zhang et al., 2020).

**TABLE 2:** Calculation of Ka/Ks for paralogous JrFats gene pairs.

Gene pair	Ka	Ks	Ka/Ks	Duplication type	Divergence time (Mya)
JrFatA1-JrFatA2	0.075	0.409	0.1833	Segmental	15.71359292
JrFatB1-JrFatB2	0.058	0.365	0.1583	Segmental	14.04206023
JrFatB5-JrFatB6	0.074	0.343	0.2159	Segmental	13.18454325

### 3.5 Promoter element analysis of JrFat gene

**FIGURE 3:**
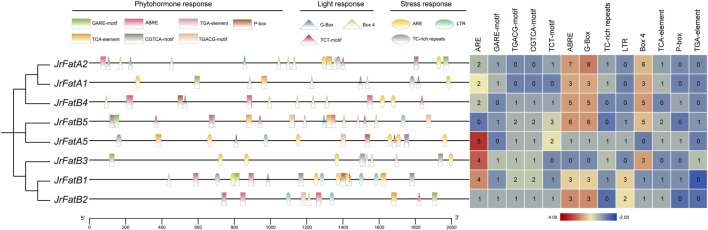
The promoter elements of *JrFats* are analyzed. *JrFats* genes are arranged according to their phylogenetic grouping, and different types of promoters are displayed in different colors and shapes. The heatmap on the right shows the statistical number of corresponding elements.

The promoter sequences, defined as the upstream 2,000 bp sequences of each JrFat gene, were analyzed to identify cis-regulatory elements (CREs). Apart from core and basic CREs, other elements were classified into three functional categories: phytohormone response, light response, and stress response (Figure 3). These categories of elements are broadly distributed across all JrFat gene promoters. Although in *JrFatB1* and *JrFatB2*, CREs tend to concentrate more towards the distal end of the promoter (between 400 and 2,000 bp), this pattern appears somewhat indistinct. Overall, there is no apparent specific or clustered distribution of CREs in the promoter regions of JrFat genes. In terms of element quantity, *JrFatB1/B3/B6* exhibit a relatively higher abundance of ARE elements, which are considered essential for anaerobic induction. Apart from these three genes, the remaining 5 *JrFatA* and *JrFatB* gene promoters contain a higher number of ABRE, G-Box, and Box4 elements. ABRE is known to be involved in abscisic acid responsiveness, while G-Box and Box4 elements are associated with plant light responsiveness. There is no significant difference in the quantity and distribution of CREs among different clades. All JrFat genes, whether FatA or FatB, appear to be extensively involved in walnut’s response to various environmental stimuli, suggesting minimal differences in regulatory expression among JrFat genes.

### 3.6 Interaction protein network analysis of JrFat gene

**FIGURE 4:**
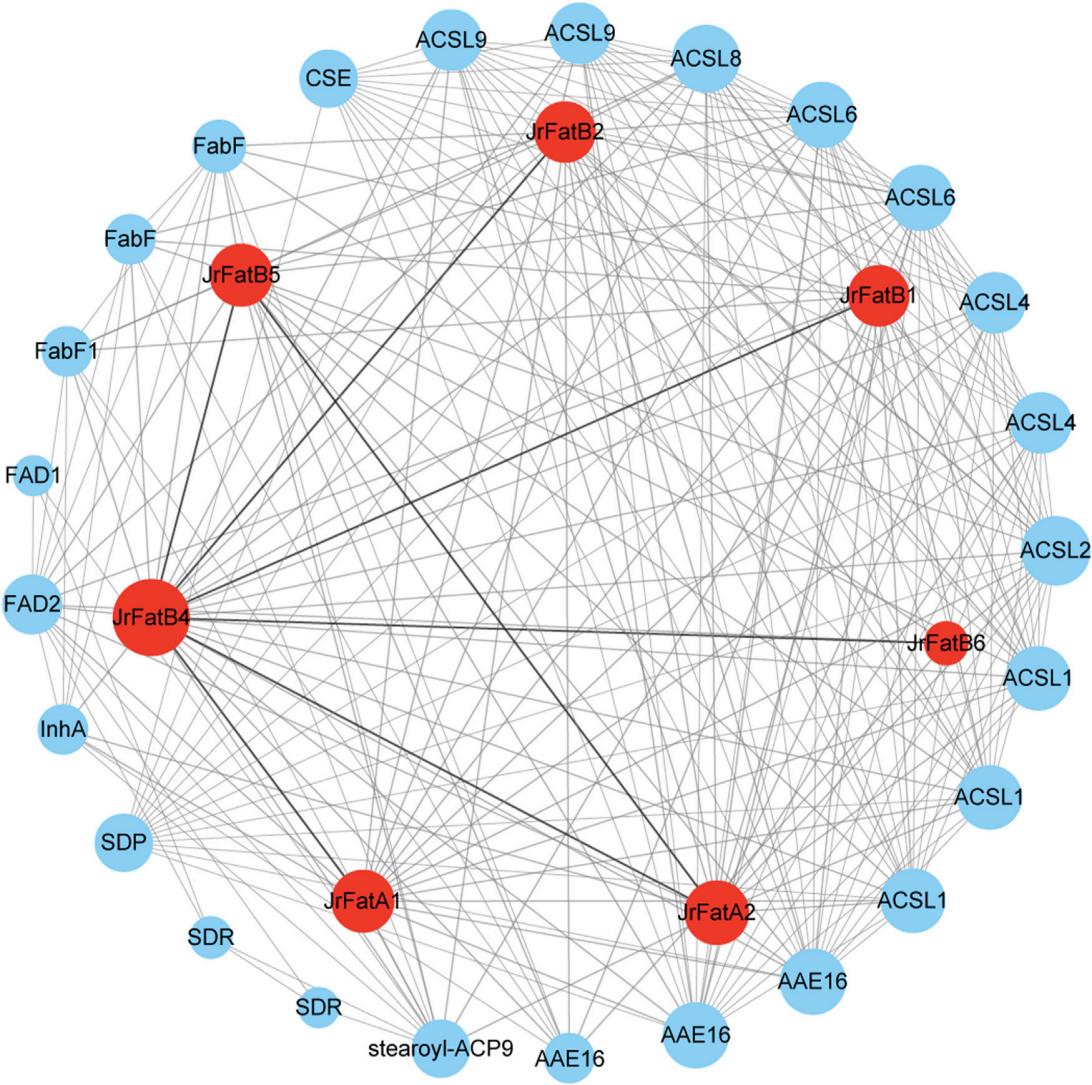
The interaction network of JrFats proteins is illustrated. JrFats proteins are represented by red nodes, other proteins by blue nodes. All interaction protein name or family were labled by the annotion of STRING. The size of the node represents the number of interacting proteins. Lines represent interaction relationships, and the thickness of the line represents the credibility of the interaction relationship.

To elucidate the function of JrFat proteins, we screened publicly available data for proteins with high-confidence interactions with JrFat proteins and constructed a protein interaction network, the names or family names of interacting proteins are annotated base STRING (Figure 4). There are seven interaction relationships among the 7 JrFat proteins, including six interactions where JrFatB4 interacts with the other JrFat proteins, and one interaction between JrFatA2 and JrFatB5, indicating JrFatB4 as the central node in the interaction network. Additionally, the 7 JrFat proteins interact with 25 other proteins, either functionally or physically. These interacting proteins mainly belong to families such as Long chain acyl-CoA synthetase-like (ACSL), acyl-activating enzyme (AAE), Beta-ketoacyl-ACP synthases (FabF), short-chain dehydrogenases/reductases (SDR), fatty acid desaturase (FAD), and Caffeoylshikimate esterase (CSE). They are widely involved in fatty acid metabolic or biosynthetic processes and are also implicated in wax biosynthetic processes, cutin biosynthetic processes, and responses to ozone, among others. These results suggest that Fat proteins may synergize with these proteins to mediate fatty acid synthesis and metabolism in different growth and developmental stages of walnuts. Notably, JrFatB3 did not exhibit any interactions with other proteins in the screening.

### 3.7 Dynamics of fatty acid content in walnut kernels

**FIGURE 5:**
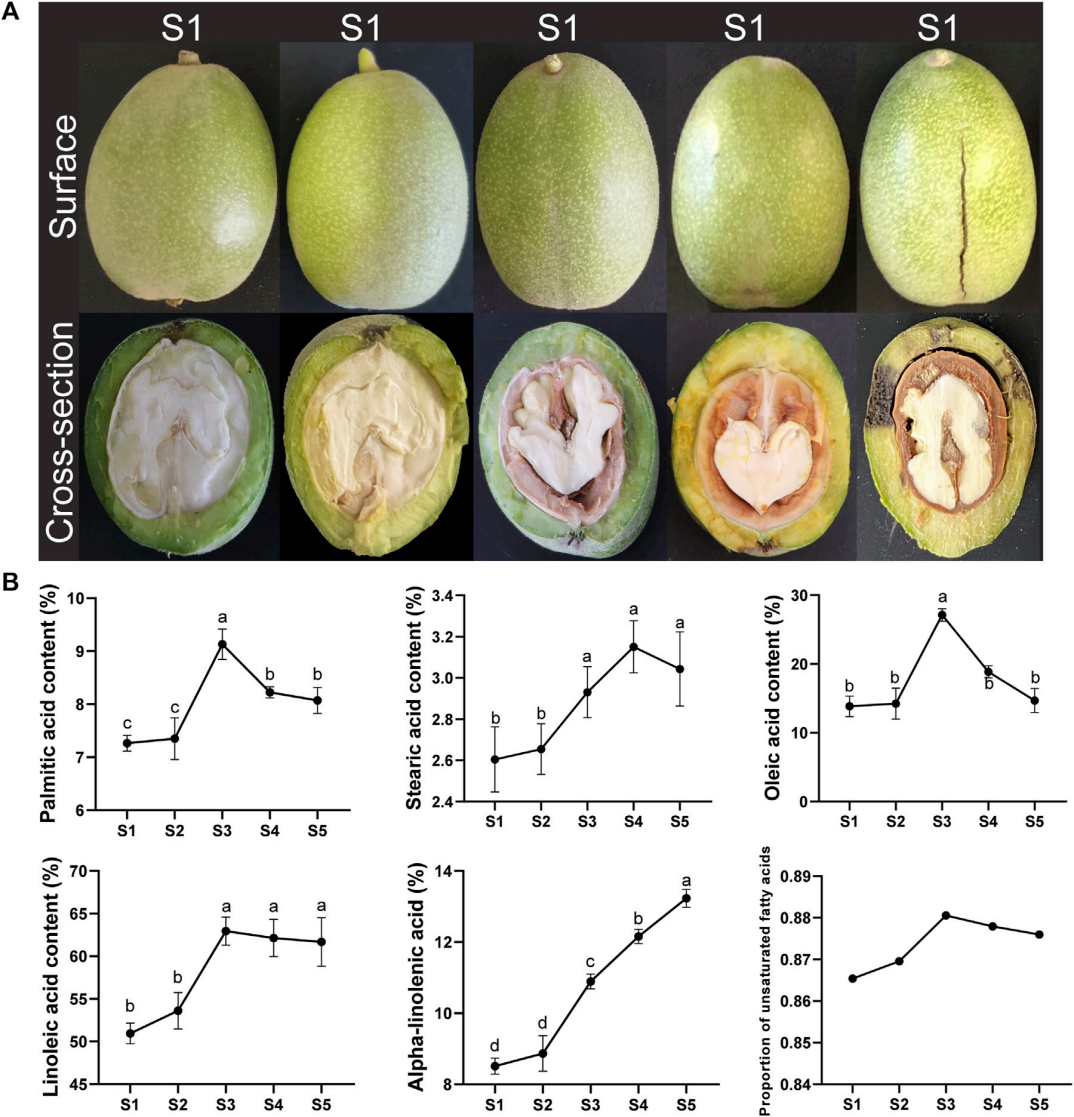
Walnut development stage and fatty acid content. **(A)** Photos of walnut samples at different developmental stages. **(B)** Line graph of fatty acid content and ratio at different developmental stages of walnuts. Lowercase letters indicate the results of significance analysis.

To clarify the changes in the main fatty acid content at different developmental stages of walnut kernels, we selected walnut fruits at five stages and measured the fatty acid content in their kernels (Figure 5). The content of two saturated fatty acids, Palmitic acid and Stearic acid, first increased and then decreased throughout the kernel development stage, and was higher at the final fruit maturity than in the early development stage. Notably, the content of Palmitic acid only increased rapidly at the S3 stage and then immediately decreased. However, the content of Stearic acid continued to rise from the S2 stage until it began to decrease at the S4 stage. Among the three unsaturated fatty acids, the content of Oleic acid showed almost the same trend as Palmitic acid, also increasing rapidly only at the S3 stage and then decreasing. The content of Linoleic acid also increased rapidly at the S3 stage, but its content remained basically unchanged in the subsequent kernels. The content of Alpha-linolenic acid continued to increase throughout the kernel development stage, especially in the late development stage (S3-S5), with significant growth at each stage. Among the five fatty acids measured, the proportion of unsaturated fatty acids fluctuated within the range of approximately 86%–88%, and fluctuated with the development of the kernel. The proportion of unsaturated fatty acids was highest at the S3 stage, at 88.05%.

### 3.8 Expression of JrFat gene family in walnut kernels

**FIGURE 6:**
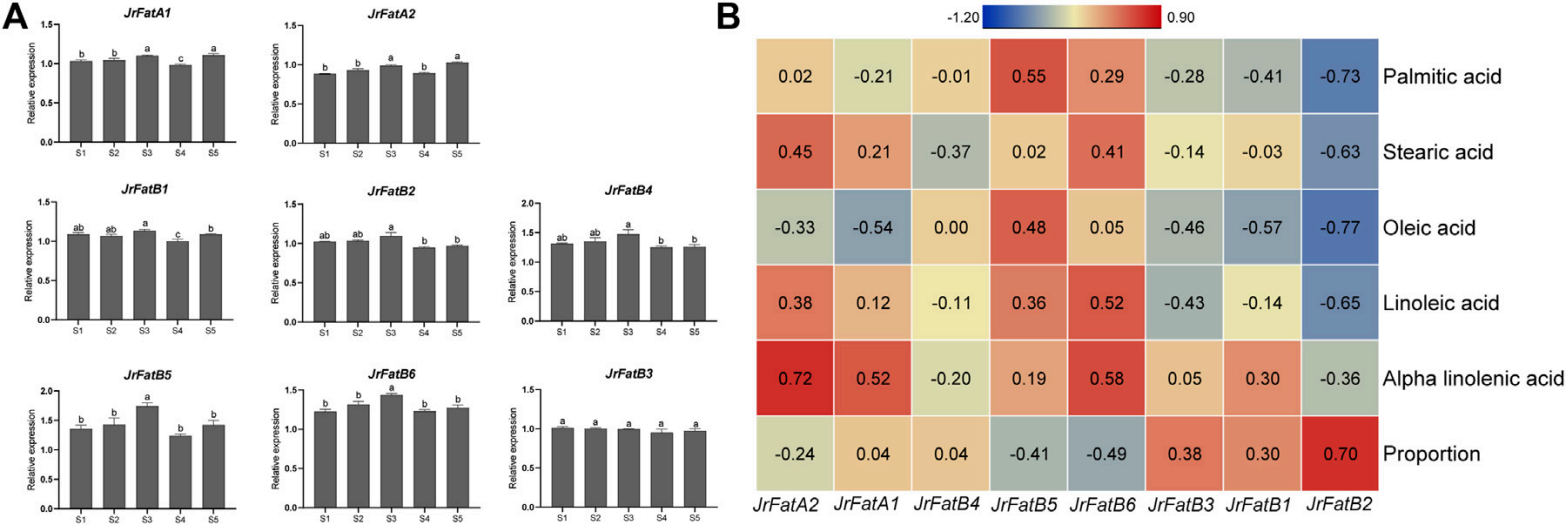
Real-time fluorescence quantification and correlation results. **(A)** Real-time fluorescence quantification of *JrFats* at different developmental stages of walnuts, lowercase letters indicate the results of significance analysis. **(B)** Heatmap of Pearson correlation between *JrFats* expression and the content of five fatty acids. The color of the heatmap indicates the degree of correlation, the redder the more positive the correlation, the bluer the more negative the correlation, and the correlation value is displayed in the corresponding square. The “Proportion” refers to the proportion of saturated fatty acids to unsaturated fatty acids.

To determine the role of the Fat gene family in the synthesis and metabolism of fatty acids in walnut kernels, we performed real-time fluorescence quantification using the same period samples. The expression of all genes was normalized according to the expression of the acting gene to avoid statistical bias caused by the five developmental stage (Figure 6A). The expression trends of *JrFatA1*, *JrFatA2*, *JrFatB5*, and *JrFatB6* genes are similar, with their expression significantly upregulated at stages S3 and S5. The difference is that *JrFatA1* is downregulated at the S4 stage while the other three genes are not. The expression trends of *JrFatB1*, *JrFatB2*, and *JrFatB4* are similar, with their expression slightly upregulated at stages S1-S3, and then downregulated at stages S4-S5. The expression of *JrFatB3* remains basically unchanged throughout the development stage. To further clarify the association between specific JrFat and fatty acid content, we calculated the correlation between the expression of 8 JrFats and the content of five fatty acids and the ratio of saturated and unsaturated fatty acids (Figure 6B). The results show that the expression of *JrFatA1*, *JrFatA2*, and *JrFatB6* has a strong positive correlation with the content of Alpha-linolenic acid, with correlations of 52%, 72%, and 58% respectively. They may positively regulate the accumulation of Alpha-linolenic acid content in unsaturated fatty acids. The expression of *JrFatB5* has a strong positive correlation with Palmitic acid, with a correlation of 55%. This gene may promote the accumulation of Palmitic acid content in saturated fatty acids. The expression of *JrFatB2* is negatively correlated with all fatty acids, with the strongest negative correlation with Palmitic acid and Oleic acid, respectively −73% and −77%. Its expression has a 70% positive correlation with the ratio of saturated and unsaturated fatty acids. This gene may mediate the conversion between different fatty acids during the development of walnut kernels. The correlation between *JrFatB1*, *JrFatB3*, and *JrFatB4* and the changes in the content of five fatty acids during all kernel development processes is relatively low.

## 4 Discussion

Walnuts are indeed a valuable nutriment, renowned for their copious content of unsaturated fatty acids, some of which are essential and must be procured through diet as they cannot be synthesized by the human body (Areias et al., 2000). These unsaturated fatty acids can enhance human blood lipid profiles, lower blood sugar levels, reduce diastolic blood pressure, and are generally considered beneficial for conditions related to metabolic syndrome (MetS), such as diabetes, cardiovascular disease, and obesity (Samei et al., 2024). Prolonged consumption of walnuts is also thought to improve intestinal health (Tindall et al., 2020), enhance the neuropsychological status of adolescents (Pinar-Martí, et al., 2023), and reduce mortality rates in middle-aged and elderly individuals (Liuet al., 2021). Moreover, the oils and fatty acids in walnuts are extensively used in other product additives, cosmetics, and the health industry (Martínez et al., 2010). The content and composition of fatty acids in walnuts indeed determine their use in food, industry, and medicine. Achieving an optimal ratio of saturated to unsaturated fatty acids is a crucial breeding target for walnuts. While some key genes affecting the fatty acid content of walnut kernels have been studied, such as the expression of JrFAD3, which is considered to be more than 99% positively correlated with the content of α-linolenic acid during 100–130 days after flowering (Liu et al., 2020), and the downregulation of JrSAD gene expression, which may cause a decrease in fatty acid and oil content levels (Liang et al., 2023), our understanding of how walnut kernels adjust the ratio between saturated and unsaturated fatty acids remains limited. Further research in this area could provide valuable insights for improving the nutritional profile of walnuts.

*Juglans regia*, a diploid species (2n = 32) with a genome size of approximately 700 Mb, is recognized for its elevated oil content. Intriguingly, it has been proposed that the quantity of Fat genes in plants is correlated with the oil content of their seeds, and is independent of the size and ploidy of the species’ genome. Species with high oil content, such as soybeans, cotton, and peanuts, contain a substantial number of Fat genes, while fewer are found in *Arabidopsis* and wheat (Zhou et al., 2021; Liu et al., 2022b; Peng et al., 2020). Despite their high oil content, walnuts only possess eight JrFat genes, fewer than in soybeans and other species. The JrFat gene family in walnuts encompasses three pairs of homologous genes: *JrFatA1*-*JrFatA2*, *JrFatB1*-*JrFatB2*, and *JrFatB5*-*JrFatB6*. These three pairs of genes exhibit a high degree of sequence consistency and good orthologous and paralogous relationships. They also exhibit the same expression trend during the development of walnut kernels, demonstrating the highly conserved evolutionary characteristics of JrFat sequences.

The species proximate to walnut in terms of evolution is Mrubra, which diverged approximately 31 million years ago (Mya). *J. regia* diverged from its related species more than 0.82 Mya. *J. regia* has undergone at least two whole-genome duplications (WGDs), which transpired approximately 130 Mya and 24 Mya (Zhang et al., 2020). The divergence time of walnut JrFats is between 13 and 15 Mya, considerably later than the second WGD, and does not coincide with the species divergence and WGD times. The expansion of JrFat may be associated with unique evolutionary events in walnut. *JrFatB3* and *JrFatB4* are particularly noteworthy. We conjecture that they are likely the fourth pair of paralogous genes, but their sequences have now undergone significant changes. *JrFatB3* has a short amino acid sequence, is the only JrFat that does not participate in the protein interaction network, and its expression remains unchanged during the development of walnut kernels, which suggests it may have lost its function or undergone pseudogenization. *JrFatB4* is even more special. It exhibits a conservative base sequence akin to *JrFatA*, and is also in the same clade as *JrFatA* on the phylogenetic tree of sequences. This is an intriguing phenomenon, which may suggest that FatA and FatB come from the same ancestral gene. Further discussion is needed on the evolution and origin of Fats genes in plants.

The Fat gene, part of the TE superfamily, plays a pivotal role in the synthesis of diverse types of fatty acids by hydrolyzing thioester bonds. Its function is largely contingent on the specific recognition of substrates. Generally, FatA recognizes unsaturated fatty acids, and FatB recognizes saturated fatty acids. However, the substrate recognition varies among different species. For instance, in Umbellularia californica, FatB1 specifically recognizes only 12:0-ACP and has low activity for other fatty acids (FAs). Its heterologous overexpression significantly augments the content of this type of fatty acid (Eccleston et al., 1996; Eccleston and Ohlrogge, 1998). In contrast, in Cuphea hookeriana, FatB1 broadly selects 14:0-ACP to 18:0-ACP as substrates (Jones et al., 1995). Interestingly, even when recognizing the same substrate, there are significant differences in activity for different FAs. For example, after *AtFatB* in *Arabidopsis* is knocked out, the content of 16:0- and 18:0-ACP both decrease by about 50% (Bonaventure et al., 2019; Liu et al., 2022b). However, when FatB is heterologously expressed in *Madhuca longifolia*, the level of C18:0 is much higher than that of C16:0 (Bhattacharya et al., 2015). In our results, the C-terminal motif of JrFat is conservative, and there is almost no difference between different JrFats. The N-terminus has three types according to the distribution of motif-9 and motif-10. JrFats with similar motifs are likely to have similar substrate recognition characteristics. We speculate that the key role played by JrFat in the synthesis of fatty acids has caused it to show typical conservative evolution, and sub-functionalization has occurred among family members to adapt to the requirements of different fatty acids in different organs and developmental stages of walnuts. The high conservation between homologous genes serves as a redundant form to mediate the same or similar functions. The test materials have different degrees of increase in fatty acid content from the hard core period, and the significant increase in JrFats gene expression is synchronized with the change in fatty acid content in time. However, from the perspective of expression difference multiples, the expression difference multiples of most JrFats in different development stages of the kernel are not substantial. This phenomenon is also observed in the overexpression experiments of soybeans and cotton (Zhou et al., 2021; Liu et al., 2022a). The biosynthesis and degradation of fatty acids are mediated by multiple factors. Although JrFat is a key enzyme in the synthesis of saturated and unsaturated fatty acids, if you want to achieve directional breeding of fatty acid content and ratio in walnut kernels, you may need to consider other node genes in the biological synthesis and metabolism network of fatty acids. The recognition of substrates by *JrFatBs* should be broad, at least their expression in walnut kernels does not show a strong correlation with Palmitic acid or Stearic acid. What is different is *JrFatA1* and *JrFatA2*. Their expression is positively correlated with the change in Alpha-linolenic acid content by more than 50% and more than 70% respectively, and the correlation with other fatty acids is lower (Wang Q. et al., 2013). Walnuts are one of the foods with the highest content and proportion of unsaturated fatty acids. One of the reasons why walnut kernels are rich in Alpha-linolenic acid may be related to the specific recognition of *JrFatAs*. *JrFatAs* may serve as a potential gene resource for increasing the content of unsaturated fatty acids in walnuts or other foods. In general, these results provide a basis for further research on the synthesis and metabolism of walnut fatty acids and the genetic analysis of JrFats gene function.

## 5 Conclusion

In this study, two *JrFatA* and six *JrFatB* members were identified from the walnut genome. These genes are composed of three pairs of highly similar motifs, evolutionarily conserved gene pairs, and two sequence-divergent members. The expression of JrFats is widely regulated by light signals and plant hormones, and interacts with FAD, ACSL, and other fatty acid synthesis-related proteins. The expression of JrFats genes increases initially and then either remains unchanged or decreases with the development of the walnut fruit. The expression trends between homologous gene pairs are similar. The upregulation of JrFats is basically synchronized with the accumulation of fatty acid content in walnuts. There is a high correlation between the expression of *JrFatA2* and the content of Alpha-linolenic acid. These results provide insights for understanding the role of JrFats genes in the biosynthesis and accumulation of fatty acids in walnuts. *JrFatA2* can be used as a potential gene resource to increase the content of unsaturated fatty acids in walnuts. This could have significant implications for improving the nutritional profile of walnuts and other foods.

## Data Availability

The original contributions presented in the study are included in the article/Supplementary Material, further inquiries can be directed to the corresponding authors.
